# Multiscale Imaging Approach for Studying the Central Nervous System: Methodology and Perspective

**DOI:** 10.3389/fnins.2020.00072

**Published:** 2020-02-07

**Authors:** Michela Fratini, Ali Abdollahzadeh, Mauro DiNuzzo, Raimo A. Salo, Laura Maugeri, Alessia Cedola, Federico Giove, Olli Gröhn, Jussi Tohka, Alejandra Sierra

**Affiliations:** ^1^IRCCS Fondazione Santa Lucia, Rome, Italy; ^2^Institute of Nanotechnology-CNR c/o Physics Department, Sapienza University of Rome, Rome, Italy; ^3^A. I. Virtanen Institute for Molecular Sciences, University of Eastern Finland, Kuopio, Finland; ^4^Museo Storico della Fisica e Centro Studi e Ricerche Enrico Fermi, Rome, Italy

**Keywords:** multimodal image coregistration, magnetic resonance image, X-ray phase contrast microtomography, multiscale imaging, brain, spinal cord, image coregistration

## Abstract

Non-invasive imaging methods have become essential tools for understanding the central nervous system (CNS) in health and disease. In particular, magnetic resonance imaging (MRI) techniques provide information about the anatomy, microstructure, and function of the brain and spinal cord *in vivo* non-invasively. However, MRI is limited by its spatial resolution and signal specificity. In order to mitigate these shortcomings, it is crucial to validate MRI with an array of ancillary *ex vivo* imaging techniques. These techniques include histological methods, such as light and electron microscopy (EM), which can provide specific information on the tissue structure in healthy and diseased brain and spinal cord, at cellular and subcellular level. However, these conventional histological techniques are intrinsically two-dimensional (2D) and, as a result of sectioning, lack volumetric information of the tissue. This limitation can be overcome with genuine three-dimensional (3D) imaging approaches of the tissue. 3D highly resolved information of the CNS achievable by means of other imaging techniques can complement and improve the interpretation of MRI measurements. In this article, we provide an overview of different 3D imaging techniques that can be used to validate MRI. As an example, we introduce an approach of how to combine diffusion MRI and synchrotron X-ray phase contrast tomography (SXRPCT) data. Our approach paves the way for a new multiscale assessment of the CNS allowing to validate and to improve our understanding of *in vivo* imaging (such as MRI).

## Introduction

Diagnosis, monitoring, surgical interventions, and treatment planning strategies of diseases in the central nervous system (CNS) strongly benefit from non-invasive imaging methods. Among all imaging techniques, magnetic resonance imaging (MRI) has become the most valuable approach in both clinical and experimental settings because of its non-invasive nature and high image quality. Conventional MRI sequences, such as T1- or T2-weighted, are extensively used to detect tissue alterations and damage during neurodegeneration, tumor growth or after injury. However, conventional MRI methods have low sensitivity for distinct early pathological processes. Other MRI methodologies, such as magnetization transfer (Filippi and Rocca, 2004, 2007; Sled, 2018), proton density (Mezer et al., 2016), susceptibility weighted imaging (Liu et al., 2015; Vargas et al., 2018), and diffusion MRI (dMRI) (Andre and Bammer, 2010; Cohen et al., 2017) are increasingly used in the imaging of the CNS. Diffusion MRI methods, such as diffusion tensor imaging (DTI) (Basser et al., 1994), can obtain tissue microstructural information and unveil tissue changes often not detectable by conventional MRI methods. Even though DTI is already an established and widely used tool, dMRI continues to develop, generating new acquisition approaches such as high-angular resolution diffusion imaging (Tuch et al., 2002), q-space imaging (Cohen and Assaf, 2002; Jensen et al., 2005) or multidimensional dMRI (Westin et al., 2016; Topgaard, 2017), and more elaborate models for data analysis (Wedeen et al., 2008; Panagiotaki et al., 2012; Zhang et al., 2012). These advanced dMRI methods have a great potential to extract intravoxel microstructural information in the CNS beyond DTI.

Other emerging MRI methods, such as quantitative susceptibility mapping (Schweser et al., 2011), can detect and discriminate between diamagnetic and paramagnetic materials, which is useful in conditions such as demyelination, calcifications or iron accumulation (Wang and Liu, 2015; Li et al., 2016; Aggarwal et al., 2018).

**FIGURE 1:**
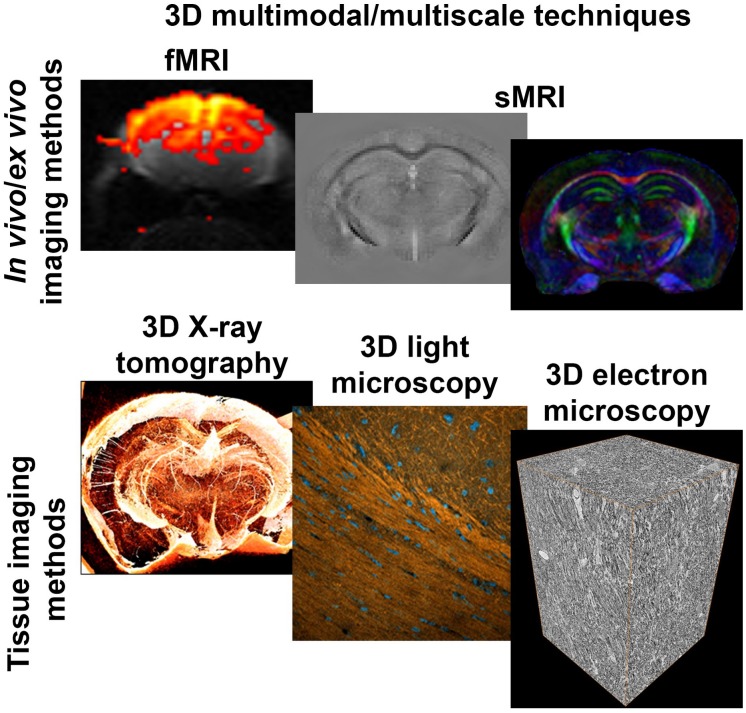
3D imaging methods. Functional and structural MRI techniques unveil tissue microstructural properties and function in health and during pathology *in vivo*. 3D multiscale tissue imaging methods offer characterization and validation of the MRI contrast *ex vivo*. DTI, diffusion tensor imaging; EM, electron microscopy; fMRI, functional MRI; LM, light microscopy; QSM, quantitative susceptibility mapping; XRPCT, X-ray phase contrast microtomography.

Despite the inherent value of structural and microstructural MRI techniques to detect abnormal tissue in the CNS, there is a lack of understanding of the origin of imaging contrast. The interpretation of MRI is a challenge because of the limited resolution, and leads us to question: *What information does a single voxel of MRI contain?* To answer this question, it is necessary to understand which tissue components dictate the MRI signal in each voxel. This level of understanding can be reached by correlating MRI contrast with underlying cellular components and characterizing the potential changes altering MRI signal. Therefore, the validation of MRI requires multimodal and multiscale imaging studies, from macro- to nanometer scale, with techniques able to show high-specificity and high-resolution tissue properties, such as cell somas, neurites, vessels, among others, and also in three-dimensional (3D) (Figure 1).

## High Resolution Imaging Methods to Validate the MRI Contrast

Traditionally, the validation of the MRI contrast has been done with *ex vivo* tissue preparations, e.g., employing histochemistry and immunohistochemistry, which are essentially two-dimensional (2D) approaches. Recent technological advances allow to image the tissue in high-resolution and in 3D with the possibility to image large tissue volumes (Figure 1). In particular, technological advances in light microscopy (LM) can image the tissue by optical sectioning of thick tissue sections (Helmstaedter et al., 2008). Confocal microscopes can image a tissue section up to 100 μm-thick (Stelzer, 2006). Tissue sections thicker than 100 μm can be imaged with more advanced techniques, such as light sheet microscopy (Weber and Huisken, 2011) or two-photon microscopy (Denk et al., 1990). Also, the resolution of LM methods have been improved in novel super-resolution techniques, such as stimulated-emission-depletion microscope reaching 40 nm planar resolution *ex vivo* (Hell and Wichmann, 1994)or structured illumination microscopy up to 100 nm resolution *in vivo* (Gustafsson, 2000). Additionally, tissue clearing techniques (Du et al., 2018), such as CLARITY (Chung et al., 2013), 3DCISCO (Ertürk and Bradke, 2013) or ScaleS (Hama et al., 2015), which make the tissue transparent and minimize light-scattering, have opened new avenues in the optical 3D imaging of large tissue coverage, e.g., whole brain.

Imaging of tissue ultrastructure can be achieved with electron microscopy (EM). Advanced EM techniques have expanded the applicability of EM by offering 3D imaging of large field-of-view of tissue (Briggman and Bock, 2012). Serial sectioning transmission EM methods can achieve nanometer in-plane resolution of large tissue sample by combining transmission EM with physical serial sectioning of the samples (Kreshuk et al., 2014). Automatic section-collection methods, such as automated tape-collection ultramicrotome (Schalek et al., 2011), avoid laborious manual cutting of thousands of sections, image alignment, and missing/distorted sections. Other serial sectioning methods, such as serial block-face scanning electron microscopy (SBEM) can reduce tissue distortions by imaging the slice prior to sectioning (Denk and Horstmann, 2004). SBEM provides 3D high-resolution images collected at the mesoscopic scale in the order of a few hundred micrometers. Focused ion beam scanning electron microscopy (Heymann et al., 2006) is another 3D block-face EM technique (Heymann et al., 2006), which can achieve 4 nm resolution in-plane.

Conventional 3D X-ray imaging techniques, such as classical radiography and tomography (the most widely used tools for imaging objects with hard X-rays), are based on absorption contrast (i.e., on the imaginary part of the refraction index) and are obtained by measuring the attenuation of X-rays through the object. Conversely, high-resolution synchrotron X-ray phase contrast tomography (SXRPCT) (Fratini, 2018) achieves the contrast by imaging the phase modulation induced by an object in a coherent beam, exploiting the real part of the complex refractive index (Fratini et al., 2015). In particular, SXRPCT enables the simultaneous 3D visualization of both dense (e.g., bone) and soft objects (e.g., soft tissue) at length scales ranging from millimeters down to hundreds of nanometers, without the use of contrast agent, sectioning or destructive preparation of the samples (Fitzgerald, 2000; Stefanutti et al., 2018). Samples for XRPCT are typically prepared by perfusing with saline solution containing heparin (50 U/ml) followed by dissection, fixation in 4% paraformaldehyde, and storage in alcohol [for more details about the sample preparation see Fitzgerald (2000) and Stefanutti et al. (2018)].

Phase contrast makes SXRPCT attractive for studies of weakly absorbing samples, both in materials and life sciences. In particular, the highest sensitivity of SXRPCT to electron density with respect to conventional CT has allowed evaluating the morphological alterations in the vascular and the neuronal networks in animal models of neurodegenerative diseases such as multiple sclerosis and Alzheimer’s disease (Bravin et al., 2012; Russo, 2017).

In the last decades, many X-ray phase contrast techniques have been developed and successfully applied. Such techniques permit to convert the phase variations of an X-ray beam, due to its interactions with the sample, in measurable intensity modulations.

A simple yet effective phase contrast method for hard X-rays is based on in-line imaging after free-space propagation, which does not require any additional optics, leading to source-limited rather than optics-limited resolution (Paganin et al., 2002). When X-rays illuminate the sample, variations in optical-path length produce slight local deviations (refraction) of the X-ray beam from its original path. When a free-space propagation distance is allowed between sample and detector, the recorded image contains the refraction information in the form of interference fringes, whose detectability depends on the coherence of the X-ray beam. Synchrotron-radiation X-ray sources provide high-photon flux X-ray beams, with a high degree of spatial coherence and allow for the possibility of providing monochromatic beams (Weitkamp et al., 2011). The high quality of the images helps optimize the algorithms used for image analysis and the 3D reconstruction. For the reconstruction of the SXRPCT volume, the first step is the phase retrieval which is applied to all projections of the tomographic measurements, using the code ANKAphase (Cedola et al., 2017; Massimi et al., 2019). The algorithm produces the projected thickness of the object, which is proportional to the refractive index decrement if the object is homogeneous. When applied to all tomographic projections, the retrieved phase maps can be fed to a standard filtered back-projection algorithm to obtain phase tomogram.

In summary, 3D high-resolution imaging methods can provide the necessary tissue information to validate the MRI contrast. LM, EM and X-ray imaging techniques offer 2D/3D high-resolution images, tissue specificity and sample coverage needed to understand the MRI signal in the healthy and diseased brain and spinal cord. Information regarding cell density or which type of cells are present in a particular area, density and orientation of axons forming fiber bundles, vascular density, shape and tortuosity, or any alteration in those tissue metrics during pathological processes can seed light into the interpretation of MRI parameters. Still, the challenge is to combine multidisciplinary and multiscale information into the same reference frame, which is the first step for analyses and quantification of areas of interest in the brain and spinal cord.

Therefore, more sophisticated co-registration tools are required to combine the MRI and tissue data into the same reference frame.

These microscopic techniques (nominal resolution of the order of one micron) could help providing a better interpretation of the images obtained with *in vivo* techniques, using higher-resolution images as a reference atlas.

Thus, from the co-registration between MRI, which has a nominal resolution of the order of 100 μm, and microscopic images, with a nominal resolution of the order of 1 μm, we can achieve information on the underlying microstructure influencing MRI contrast, such as vessels and neurons.

## 3D dMRI/SXRPCT Co-Registration Method of the Mouse CNS

**FIGURE 2:**
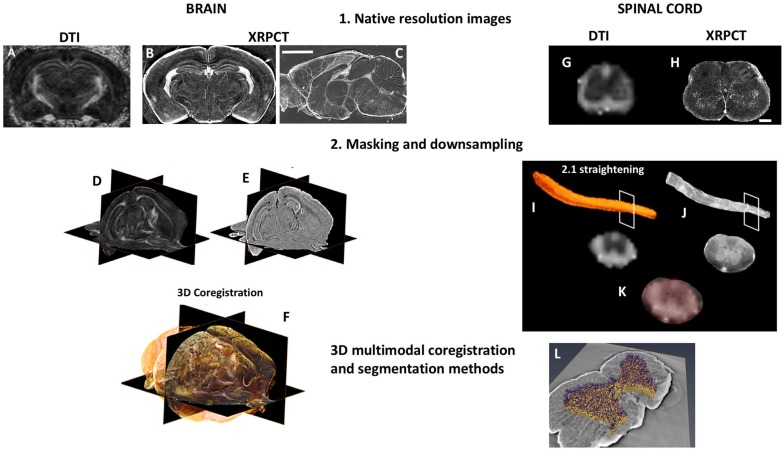
3D dMRI/XRPCT co-registration of the mouse brain and spinal cord. From the same subject, 3D dMRI and XRPCT image datasets were obtained. The native resolution of dMRI was 125 μm^3^ isotropic resolution **(A,G)**, where XRPCT image spatial resolution was 6 μm **(B,C,H)**. The scale bar is 500 μm in the brain and 200 μm in the spinal cord. To facilitate the co-registration, masking of both FA and XRPCT images **(D,I)** and down-sampling of XRPCT images to the image resolution of dMRI maps **(E,J)** were performed. In the case of the spinal cord, we performed the straightening of the whole spinal cord. In the background of panels **(F,K)**, grayscale images represent FA map of the mouse brain, and yellow rendering in the brain and red in spinal cord show the XRPCT data. **(L)** Vascular segmentation of the co-registered spinal cord.

Here, we briefly describe a pipeline to co-register dMRI and SXRPCT data from the C57BL/6J mouse brain and spinal cord acquired from the same animal (Figure 2). *Ex vivo* DTI was acquired with a 9.4 T scanner using segmented spin-echo EPI (TE = 32 ms, TR = 1 s, *b*-value = 3000 s/mm (Filippi and Rocca, 2004), 42 diffusion directions, and 125 μm-isotropic resolution). We generated fractional anisotropy (FA) and directionally encoded colored maps. SXRPCT data was acquired at ID17 beamline at the European Synchrotron Radiation Facility (ESRF) in Grenoble, France. Free-space propagation method, beam energy = 34 keV, CCD pixel size = 3 μm, detector-sample distance ≈ 2.3 m, and samples loaded in a cell filled with agar-agar were used. As the different 3D imaging techniques require different holders, the curvature of the images may differ from each other to an unknown degree. We propose two potential solutions to the co-registration problem. In the first approach, we segmented 3D images acquired with both techniques, co-registered the images to each other, and fine-tuned using non-linear co-registration tools such as SyN (Avants et al., 2008). This approach works as long as the curvature of the images acquired by MRI and SXRPCT is reasonably similar. While this assumption is generally true with brain samples, this is typically not the case for the spinal cord. This required the development of a second approach, wherein, first of all, we skeletonized both of the segmented images. The resulting skeletons were then straightened, and the image planes perpendicular to the skeletons were moved using the translations and rotations derived from the straightening process.

### 3D dMRI/SXRPCT Co-registration Method of the Mouse Brain

First, SXRPCT images were low pass filtered to avoid aliasing, and we down-sampled the images to roughly match the dMRI resolution [125 μm (Sled, 2018)]. Then, dMRI images were brain-masked using FSL’s BET (Smith, 2002) on the B0 image (Figure 2D). Since no suitable tools for automatically masking SXRPCT images are presently available, we used a combination of custom-made MATLAB routines for intensity thresholding, morphological operations, and manual fine-tuning for this task. The brain boundaries showed marginal ring artifacts due to different material density at the interface. An initial brain outline mask was produced using intensity thresholding and including low and high intensities into the mask to reduce the artifact. The mask was then dilated with a 3D circle-shaped structuring element (MATLAB imdilate; radius = 2 voxels) to close the borders and hole-filled using 2D operations (MATLAB-imfill) on the single slices. This was followed by erosions (MATLAB-imerode) with the 3D ball-shaped structuring element until parts of the mask outside of the brain disappeared. The mask was finally dilated to reach the edges of the brain volume (Figure 2E). Since FA contrast resembled the contrast in SXRPCT images in some regions, such as the corpus callosum, hippocampus proper and ventricles, we were able to co-register the FA images to the corresponding SXRPCT using advanced normalization tools (ANTs) (Avants et al., 2009). In addition to affine co-registration, we used non-linear diffeomorphic registration SyN (Avants et al., 2008) with Mattes metric (Figure 2F) to compensate for possible small sample alterations due to different preparations and susceptibility-induced distortions in the dMRI imaging.

Our analysis yielded a good anatomical agreement between dMRI and SXRPCT images after co-registration. After the 3D co-registration, the native resolution of SXRPCT images can be restored allowing a more advanced analysis and validation of the dMRI maps.

Therefore, a 3D high-resolution micro-imaging technique is required in order to delineate simultaneously the complex vascular organization, down to the smallest capillaries, and the neuronal and axonal morphology in a large volume of tissue (i.e., the same volume of the MRI scanning) This is a fundamental step toward a better understanding of the neuro-vascular coupling.

### 3D dMRI/SXRPCT Co-registration of the Mouse Spinal Cord

While existing co-registration tools could be used forthe brain, they do not work for the spinal cord and different template and toolbox have been proposed (De Leener et al., 2017; Cohen-Adad, 2018). The geometry of spinal cord requires more sophisticated tools to co-register datasets into the same reference frame, and in particular the coarse invariance for axial translations is a significant challenge for proper co-registration. Therefore, we devised a potential strategy, as described below, based on a combination of MATLAB routines to obtain a good quality co-registration between dMRI and SXRPCT images of the spinal cord. Since the bones, i.e., vertebrae, were removed prior to the imaging, the location of the intervertebral discs based on image intensity was not possible. Therefore, both the SXRPCT and dMRI data were manually labeled at two consensus reference coordinates, e.g., close to the sample’s extremities. These reference points were necessary to insert the images of the two different modalities in the same space, regardless of the image resolution. We use the spinal cord toolbox (SCT) (De Leener et al., 2017) for spinal cord straightening (Figures 2I,J).

Synchrotron X-ray phase contrast tomography images were first intensity-adjusted saturating top and bottom 0.3% and down-sampled using volumetric nearest-neighbor interpolation (Figure 2). The scaling value for down-sampling was chosen to maximize the image gradient at the sample-medium interface on selected slices in the xy-plane. Then, the contours of the spinal cord were determined in the volume using the “*approximate Canny* method” for 3D-edge-detection (Canny, 1987) (the approximate Canny method uses two thresholds to detect strong and weak edges). Image intensity threshold was calculated using Otsu’s method (Otsu, 1979). Two different approaches were used to determine the spinal cord position on the SXRPCT images. The first was based on the center of mass of contour images determined on slices in the xy-plane, which yielded spinal cord centerlines. The second was based on filling of edge images using three-dimensional seeded region growing, which yielded spinal cord binary masks. The original SXRPCT stack and either the spinal cord centerline or the binary mask were then fed to the SCT straightening algorithm (De Leener et al., 2017). The SXRPCT binary mask and DTI images were finally then co-registered using ANTs (Figures 2K,L). This approach can be especially useful with parametric maps derived in the original image reference frames that could be transformed into the straightened form. This approach is also robust with respect to different curvatures in the images and, most importantly, it allows us to place all the spinal cord images from different individuals into the same reference frame. In addition, with XRPCT it is also possible to extract information simultaneously about the neuronal and vascular networks.

The potential outcomes of advancing these methods are great, enhancing our basic understanding of healthy human CNS function, and improving our ability to accurately diagnose and treat injury and disease and predict treatment outcomes.

The co-registration methods presented here open new possibilities to obtain complementary information to validate MRI. Future directions are to include other histological modalities, as 3D LM or EM data as well as other MRI modalities, into the pipeline. Also, new analysis methods, such as segmentation and quantification of individual cellular components (Abdollahzadeh et al., 2019a, b; Figure 2L), allow to extract more specific morphological information for the evaluation of areas under investigation. A multiscale and multidisciplinary approach as proposed in this article paves new ways to study the brain and spinal cord, and more importantly, to better understand the non-invasive information given by *in vivo* MRI.

## Discussion

Magnetic resonance imaging has become one of the most powerful tools in neuroscience research, with a wide range of applications in both clinical practice and research settings. However, the interpretation of highly complex MRI contrasts remains a daunting task, which would benefit tremendously from the progress of multimodal and multiscale imaging approaches.

Synchrotron X-ray phase contrast tomography, LM, or EM can perform 3D imaging of post-mortem CNS tissue displaying, e.g., from the architecture of the neuronal and vascular network up to a single neuronal soma. These methods can be useful to validate *in vivo* imaging techniques using the high-resolution images as a reference (Schulz et al., 2012; Gangolli et al., 2017; Cohen-Adad, 2018). In particular, LM and EM offer high-resolution and specificity to visualize different cellular substrates, which have already exploited for the microstructural MRI validation (Mac Donald et al., 2007; Budde et al., 2011; Salo et al., 2017; Cohen-Adad, 2018; Duval et al., 2019). In the recent years, LM and EM have been developed into 3D imaging techniques generating new ways for the MRI validation (Khan et al., 2015; Schilling et al., 2016, 2018; Salo et al., 2018; Lee et al., 2019). On the other hand, SXRPCT offers the possibility to study the tissue in 3D without sample sectioning and specific sample preparation (Fitzgerald, 2000). SXRPCT allows both the assessment of the overall 3D morphology of the sample and fine computing sectioning, as thin as 130 nm. SXRPCT has been applied in the imaging of the 3D distribution of vasculature and the single elements of the neuronal network in healthy and pathological CNS (Bravin et al., 2012; Stefanutti et al., 2018). The combination of the MRI with high-resolution SXRPCT can, in principle, allow the assessment of the tissue morphology and the segmentation of different anatomical structures (Schulz et al., 2012) thus permitting to shed light on pathological alterations.

Co-registration methods, as the one presented here, allow to gather information from complementary imaging methods at different length scales spanning from the macroscopic to the nanometric level. The application of these techniques to *ex vivo* brain and spinal cord, for example, allows quantifying microstructural alterations in diseased subjects, and it has the potential to lead to a better understanding of the relationship between structure and function in the CNS. One of the major limitations of the co-registration using different imaging modalities on the same sample is the tissue preparation (Fitzgerald, 2000). In this work, the tissue was fixed with paraformaldehyde, which only induces a minor tissue shrinkage. Other methods, such as LM or EM, require that the tissue be stained, resulting in a more significant shrinkage that can complicate the co-registration process. Prospectively, the added value of a multimodal and multiscale imaging can be greatly increased by further improvements in co-registration approaches such as those outlined here.

## Data Availability Statement

The raw data supporting the conclusions of this article will be made available by the authors, without undue reservation, to any qualified researcher.

## Ethics Statement

All animal procedures were carried out under licenses that have been approved by the Animal Ethics Committee of the Provincial Government of Southern Finland and in accordance with the guidelines of the European Community Council Directives 86/609/EREC.

## Author Contributions

MF and AS contributed to the concept and study design. AA, MD, RS, and LM performed the analyses. All authors have contributed to the interpretation the data, wrote the main manuscript text, and reviewed the manuscript.

## Conflict of Interest

The authors declare that the research was conducted in the absence of any commercial or financial relationships that could be construed as a potential conflict of interest.

## Abbreviations

2D: two-dimensional
3D: three-dimensional
ANTs: advanced normalization tools
CNS: central nervous system
dMRI: diffusion magnetic resonance imaging
DTI: diffusion tensor imaging
EM: electron microscopy
ESRF: european synchrotron radiation facility
FA: fractional anisotropy
LM: light microscopy
MRI: magnetic resonance imaging
SCT: spinal cord toolbox
SXRPCT: synchrotron X-ray phase contrast tomography.
